# Evaluation of selective bone scan staging in prostate cancer – external validation of current strategies and decision-curve analysis

**DOI:** 10.1038/s41391-022-00515-8

**Published:** 2022-03-14

**Authors:** Mrunal D. Hiwase, Alex Jay, Norma Bulamu, Johnathan Teh, Felix Paterson, Ganessan Kichenadasse, Andrew D. Vincent, Michael O’Callaghan, South Australian Prostate Cancer Clinical Outcomes Collaborative (SA-PCCOC), Tina Kopsaftis, Scott Walsh

**Affiliations:** 1 University of Adelaide, Adelaide Medical School, Adelaide, SA Australia; 2 Department of Surgery, Central Adelaide Health Network, Adelaide, SA Australia; 3 Flinders Medical Centre, Urology Unit, Adelaide, SA Australia; 4 Health Economist, Flinders Health and Medical Research Institute, Flinders University, Adelaide, SA Australia; 5 Northern Adelaide Health Network, Adelaide, SA Australia; 6 Nuclear Medicine Physician and Radiologist, Dr Jones and Partners Radiology and Flinders Medical Centre, Adelaide, SA Australia; 7 Flinders Centre for Innovation in Cancer, Flinders Medical Centre/Flinders University, Bedford Park, SA 5042 Australia; 8 Freemasons Centre for Male Health and Wellbeing, University of Adelaide, Adelaide, SA Australia; 9 Department of Urology, Flinders Medical Centre, Bedford Park, SA Australia; 10 South Australian Prostate Cancer Clinical Outcomes Collaborative (SA-PCCOC), Adelaide, SA Australia; 11 Envido (Digital Health Insights), Adelaide, 5000 SA Australia

**Keywords:** Prostate cancer, Outcomes research, Cancer epidemiology

## Abstract

**Background:**

Recommendations for staging newly diagnosed prostate cancer patients vary between guidelines and literature.

**Methods:**

Our objective was to validate and compare prediction models selecting newly diagnosed prostate cancer patients for bone scan staging. To achieve this, we validated eleven models in a population-based cohort of 10,721 patients diagnosed with prostate cancer between 2005 and 2019. The primary outcome was net-benefit. This was assessed at different balances of conservatism and tolerance, represented by preference ratio and number-willing-to-test (NWT). Secondary outcomes included calibration slope, calibration-in-the-large (intercept), and discrimination measured by Area-under-the-receiver-operator-characteristics curve (AUC).

**Results:**

For preference ratios less than 1:39 (NWT greater than 40), scanning everyone provided greater net-benefit than selective staging. For preference ratios 1:39 to 3:97 (NWT 33–40), the European Association of Urology (EAU) 2020 guideline recommendation was the best approach. For preference ratios 3:97–7:93 (NWT 14–33), scanning EAU high-risk patients only was preferable. For preference ratios 7:93–1:9 (NWT 10–13), scanning only Gnanapragasam Group 5 patients was best. All models had similar fair discrimination (AUCs 0.68–0.80), but most had poor calibration.

**Conclusions:**

We identified three selective staging strategies that outperformed all other approaches but did so over different ranges of conservatism and tolerance. Scanning only EAU high-risk patients provided the greatest net-benefit over the greatest range of preference ratios and scenarios, but other options may be preferable depending upon the local healthcare system’s degree of conservatism and tolerance.

## Introduction

Prostate cancer mortality is highly dependent upon stage of disease, and assessment of metastatic diseases at prostate cancer diagnosis is critical for adequate treatment planning and selection between potentially morbid treatment options. Bone scan staging remains the most widely available tool for quantifying metastatic burden and most supported for basing treatment decisions upon [1, 2]. Yet, recommendations on which patients to scan vary between guidelines and literature. Guideline recommendations are reported as weak [1] or based only upon expert opinion and grade 2A–C evidence [2, 3]. Recommendations from the primary literature were developed in small selective cohorts [4–6] often based upon insensitive performance markers (like negative predictive value), infrequently externally validated and if validated, done so in small selective cohorts [7–19]. Head-to-head comparisons of strategies are also limited [7, 13–16].

Decision curve analysis offers a novel approach to evaluate these strategies and compare them at various levels of conservatism (preference to avoid missing a positive scan) and tolerance (preference to limit number of people scanned). This approach compares strategies on net-benefit, which considers the positive scans detected by a particular strategy and the number of people scanned with it, weighting these two results by the conservatism and tolerance of the preferred strategy type.

We use decision curve analysis to review and validate strategies for bone scan staging in patients with newly diagnosed prostate cancer, comparing them against major clinical guidelines. The aim is to identify optimal strategies for bone scan staging in newly diagnosed prostate cancer patients.

## Subjects and methods

### Identifying models used for selective bone scan staging

Models were chosen from published literature and guidelines and validated in the South Australian Prostate Cancer Clinical Outcomes Collaborative (SA-PCCOC) database. A model was defined as any allocation of bone scan positivity risk to a group of newly diagnosed prostate cancer patients based on a predictor(s). MEDLINE and EMBASE databases were searched for models using keywords: Prostate Cancer, Metastases, Prediction, Staging, Screening and Imaging (with related terms) and an English-only limit. Titles and abstracts were screened for relevance. Abstract-only records and reviews were manually excluded. Articles containing models predicting bone scan positivity, using common clinical predictors, were further assessed. Those using tests not routinely available (circulating tumour cells, cell-free DNA and similar) were excluded. Common predictors included serum Prostate Specific Antigen, Tumour stage and Gleason score (GS) at diagnosis. We used the Prediction model Risk Of Bias ASsessment Tool (PROBAST) tool [20] for quality assessment.

### Validation cohort

The cohort comprised of all patients diagnosed between 1 January 2005 and 26 May 2019 in the SA-PCCOC registry. This registry captures more than 90% of prostate cancer patients diagnosed in South Australia, collecting data on disease characteristics at diagnosis, initial treatment type, cause of death, time to biochemical recurrence and more. Patients are retained unless they opt-out of data collection. Survival data is obtained from the births, deaths and marriages registry and is available for all patients. Only patients diagnosed before 2005 or without a diagnosis date were excluded.

### Model outcome

Bone scans performed within 20 weeks of histological diagnosis were considered staging scans [21]. Indeterminate scans were reclassified as positive or negative using subsequent imaging and clinical information. Where further classification was unachievable, results were imputed.

### Model predictors

Most models used serum prostate-specific antigen (PSA), tumour (T) stage and/or GS as predictors. For validation, PSA prior to treatment and closest to diagnosis were used for “PSA at diagnosis”. If all PSA levels on record were post-treatment, PSA was set as unknown and imputed. T-stage was assessed by physical exam at diagnosis. GS was based on diagnostic biopsies.

### Ethics

The SA-PCCOC research committee approved use of de-identified data, having permission to authorize this from the Southern Australian Clinical Human Research Ethics Committee. This study was performed in accordance with the Declaration of Helsinki 2013.

### Statistical methods

#### Calibration

Calibration slope and calibration-in-the-large (calibration intercept) were assessed to gauge accuracy of model predictions of the risk of bone scan positivity. These were calculated by fitting logistic regressions of observed risk of bone scan positivity against predicted risk [22]. Calibration-in-the-large was similarly calculated with slope fixed at one [22]. These analyses were performed in each imputed dataset and pooled using Rubin’s rules [23]. Ideal calibration slope is one and calibration-in-the-large is zero [22]. Where predicted risk was not specified for a model risk group, the rate of bone scan positivity in the model’s development study was taken as predicted risk (Supplementary Table 6). Calibration was not calculated for guideline models, which did not report numeric predicted risks.

#### Discrimination

Area-under-the-receiver-operator-characteristics curve (AUC) was used to summarize model ability to discriminate between patients with a positive and negative bone scan. AUC was interpreted in accordance with Hosmer et al. [24].

#### Decision curve analysis

Decision curve analysis was used to compare the net-benefit of models at different scanning thresholds (staging strategies) over varying degrees of conservatism (preference to avoid missing disease) and tolerance (preference to scan fewer people) [25]. Traditionally, varying degrees of conservatism and tolerance (“preference”) are reflected in the *x*-axis of decision curves as a probability threshold (*p*_*t*_)—the point at which the user believes intervention is appropriate. To avoid confusion between model thresholds and *p*_t_, we used the alternative measure of preference ratio [25] and number-willing-to-test (NWT). A preference ratio of 1:99, in this context, represents a belief that scanning one hundred people to capture one positive bone scan is reasonable [25], and a *p*_t_ of 0.01 and NWT of 100. A preference ratio of 1:9 was the upper limit of preference assessed, as it represents a willingness to scan at least ten patients to capture one positive bone scan—a number we felt was universally acceptable.

Continuous and categorical models were presented differently. As categorical models provide qualitative rather than quantitative predictions, they had fewer potential decision thresholds. They were presented as fixed strategies, akin to the presentation of a “test” in Vickers et al. [25], with each potential threshold from a categorical model displayed as a straight-line across the range of preference ratios assessed (equation in Supplementary 2). Continuous models were presented as both decision-analysis curves (demonstrating potential outcomes of using any threshold in that model) and straight lines for the fixed strategies their source articles recommended. Strategies with higher net-benefit were considered higher performing, the magnitude of this difference being irrelevant [25].

### Missing data

Missing data were multiply imputed using chained equations (*mice* package [26]). Based upon analyses in Supplementary 1, reasons for missingness were felt well explained and correlated to prostate cancer-specific overall survival, initial treatment, treatment in a public or private setting, biopsy type and disease factors, allowing the missing-at-random assumption. We imputed one hundred datasets, each with one hundred iterations, and pooled results using Rubin’s rules [23]. Kaplan–Meier curves were used to compare survival in patients with imputed positive bone scans to those with observed positive scans, and likewise for imputed negative bone scans (Supplementary 1).

All statistical analyses were performed using R version 3.4.2 [27].

## Results

### Validation cohort

**Fig. 1:**
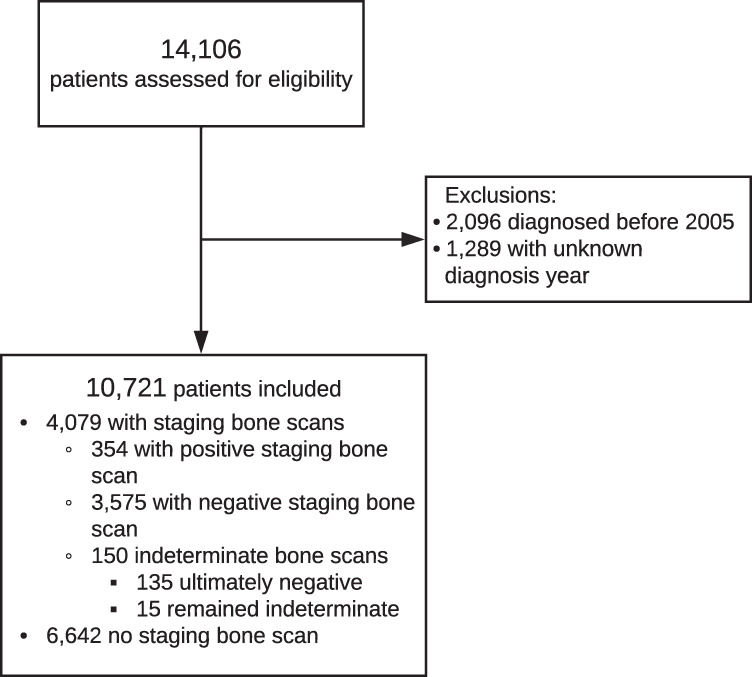
Selection of validation cohort. Flow diagram demonstrating cohort selection process, excludion criteria and cohort breakdown for selective staging strategy validation.

The cohort is comprised of 10,721 consecutive men newly diagnosed with prostate cancer (Fig. 1), 4,079 of whom had a staging bone scan and 354 (8.7%) of which were positive (Table 1). As expected, patients with positive scans had poorer survival and higher GSs, PSA at diagnosis, clinical T-stage and percent positive cores on biopsy than those with negative scans. There were 150 indeterminate bone scans (3.6%, 150/4079), the majority of which were (*n* = 135) were subsequently reclassified as negative based on follow-up imaging and data. The remaining fifteen were imputed. 6642 patients had no staging bone scan result in our database. These patients had lower GS and T-stage than patients with staging bone scans on record, were more often treated in the private setting (Supplementary Table 1) and had better survival (Supplementary Fig. 2). This points towards two main mechanisms of missing data, selective use of bone scan staging (in patients thought to be at “higher risk” as per previous clinical guidelines) or restricted access to data in privately treated patients. As the difference in survival between patients with and without bone scan minimizes with stratification by risk group (Supplementary Fig. 3 and Supplementary Table 2), there is strong support for this mechanism of missingness and thus our choice of imputation model. Supplementary 1 confirms reliability of imputations. Survival was almost identical in patients imputed with a positive bone scan, compared to those with a known positive scan, and likewise for patients imputed with negative scans (Supplementary Fig. 1). Post-imputation cohort characteristics (Supplementary Table 3) show that distribution of disease stage and incidence of metastatic disease was similar in our cohort to the SEER database [28].

**Table 1:** Characteristics of the validation cohort prior to multiple imputation.

	Overall	Staging bone scan (BS) result		No staging BS on record
		Negative	Positive	
*n*^a^	10,721	3710	354	6657
Age at diagnosis (mean [range])	67.90 [34, 98]	68.27 [41, 94]	72.71 [44, 95]	67.42 [34, 98]
Gleason Sum Score (%) Missing data: 2.4%				
≤6	3817 (36.5)	1051 (28.7)	35 (10.5)	2727 (42.2)
3 + 4	2777 (26.5)	1056 (28.9)	36 (10.8)	1684 (26.1)
4 + 3	1696 (16.2)	719 (19.7)	46 (13.8)	928 (14.4)
≥8	2174 (20.8)	832 (22.7)	217 (65.0)	1122 (17.4)
Percent Positive Biopsy Cores (median [IQR]) Missing data: 17.1%	33.33 [16.67, 56.25]	40.00 [23.08, 61.11]	80.38 [50.00, 100.00]	30.77 [16.00, 50.00]
PSA at diagnosis (median [IQR]) Missing data: 40.1%	8.20 [5.80, 13.00]	9.10 [6.40, 15.00]	63.00 [15.30, 222.80]	7.47 [5.36, 11.00]
Clinical T-stage (%) Missing data: 76.6%				
T1a–c	1593 (63.6)	681 (58.2)	33 (28.9)	876 (71.9)
T2a–c	719 (28.7)	419 (35.8)	30 (26.3)	270 (22.2)
T3a–c	127 (5.1)	62 (5.3)	19 (16.7)	45 (3.7)
T4	67 (2.7)	8 (0.7)	32 (28.1)	27 (2.2)
Clinical nodal stage (%) Missing data: 76.0%	128 (5.0)	45 (3.7)	28 (47.5)	54 (4.2)
5 year estimated survival [95% confidence interval] (%)	87 [86, 88]	88 [87, 90]	48 [42, 54]	88 [87, 89]

^a^Fifteen people with indeterminate bone scans, could not be classified as positive or negative scans. They are not included in the above table (accounting for the differences in overall cohort and sum of subgroups presented above), but outcomes for them were imputed with patients missing a bone scan result. Their personal and disease characteristics were not substantially different to the overall cohort.

*PSA* prostate-specific antigen, *IQR* interquartile range.

### Model identification

Thirteen distinct models were identified from the guidelines and literature search (Supplementary Fig. 4): EAU 2020 risk strata [29], AUA 2018 risk strata [3], NCCN 2019 risk strata [30], Ho [31], Wang [32], Chybowski [33], Briganti [4], O’Sullivan [34], Lai [5], ISUP [7], Gnanapragasam [7], Wang 2 [35] and Lorente [36]. Two could not be validated (Wang 2 [35] and Lorente [36]) as they used serum alkaline phosphatase (not recorded in the database). Three provided continuous estimates of risk based on logistic regression (Ho [31], Wang [32] and Chybowski [33]), while others categorized patients as low, intermediate, high-risk or similar based upon common clinical thresholds [3, 5, 7, 29, 30, 34] or classification-and-regression-training [4]. Thresholds recommended from these models were used to select for bone scanning (Table 2).

**Table 2:** Model details and characteristics of sources.

Tool	Country	Number scanned	Positive bone scans (%)	Model	Predictors	Recommended inclusion criteria for bone scan staging
Chybowski et al. 1991 [33]	USA	521	71 (14%)	Graph providing continuous prediction of the probability of a positive bone scan	PSA	Strategy 1: Scan when PSA > 10 Strategy 2: Scan when PSA > 20
O’Sullivan et al. 2003 [34]	England	420	67 (16%)	Categorizes patients at low or high risk	PSA, T-stage and GS	Scan when PSA > 20, T-stage 4, or GS ≥ 4 + 3
Briganti et al. 2010 [4]	Italy	853	24 (3%)	Categorizes patients risk as low, intermediate or high risk:	PSA, T-stage and GS	Scan when GS ≥ 8, or PSA > 10 in T2-3 disease
Lai et al. 2011 [5]	China	116	34 (29%)	Divides patients into PSA categories, with risk increasing with PSA. Claims patients at high risk if PSA ≥ 10	PSA	Scan when PSA > 10
Ho et al. 2013 [31]	Malaysia	258	93 (36%)	Equation providing continuous prediction of probability	PSA, Nodal status on cross-sectional imaging	Scan when PSA > 10 or suspected lymph node involvement on CT/MRI
Wang et al. 2013 [32]	China	488	65 (13%)	Equation providing continuous prediction of probability, with an adjusted equation to facilitate decisions (calculating D)	PSA, T-stage and GS	Scan when *D* < 0, where *D* = –6.40 + 2.39Tstage4 + 0.87 ln(PSA + 1) + 0.93GS + 2.169, where Tstage4 = 1 if T4 or 0 if less, and GS = 1 if Gleason score ≥4 + 3 or 0 if less
Gnanapragasam [6] repurposed by Thurtle et al. 2016 [7]	UK	438	37 (8%)	Categorizes patients into groups based as described in Gnanapragasam et al., 2016 [6]	PSA, mpMRI defined T-stage, ISUP grade group	Strategy 1: Scan when Gnanapragasam Group 3 or higher i.e. GS ≥ 4 + 3, or patients with any two of: GS 3 + 4, PSA 10–20 or T1-T2 Strategy 2: Scan when Gnanapragasam Group 4 or higher i.e. GS ≥ 8 or PSA > 20 or T≥T3
ISUP repurposed by Thurtle et al. 2016 [7]	UK	438	37 (8%)	Categorizes patients by ISUP grade grouping system	ISUP grade group	Scan when Gleason Grade Group 3 or higher
AUA guideline 2018 [3]	–	–	–	Categorizes patients by AUA risk stratification system	PSA, T-stage, GS, Percentage positive cores, PSA density^a^	Scan when unfavourable intermediate risk and higher i.e. PSA > 20, PSA > 10 when GS 3 + 4, GS ≥4 + 3, T≥ T2b
EAU guideline 2020 [29]	–	–	–	Categorizes patients by EAU risk stratification system	PSA, T-stage and GS	Scan when intermediate risk with GS 4 + 3 disease or high risk i.e. PSA > 20, T≥T2c or GS≥4 + 3
NCCN guideline 2019 [30]	–	–	–	Categorizes patients by NCCN risk stratification system	PSA, T-stage, GS, Percentage positive cores, PSA density^a^	Scan when unfavourable intermediate risk or higher i.e. T ≥T3, PSA > 20, GS ≥ 8, or any of T2b-T2c/GS 7/PSA 10–20 AND percentage positive cores on biopsy ≥50%

EAU, AUA and NCCN guidelines are refinements of the D’Amico classification of prostate cancer, which was originally designed to predict risk of biochemical recurrence following radiotherapy and has not been extended to other outcomes. Refinements to the D’Amico classification are based on multiple studies showing added value of other markers (PSA density, percent positive cores and so on). Hence, there is no specific development study for these models.

*GS* Gleason score, *PSA* prostate-specific antigen measured in ng/mL, *mpMRI* multiparametric MRI, *ISUP* International Society of Urological Pathology, *AUA* American Urological Association, *EA*U European Association of Urology, *NCCN* National Comprehensive Cancer Network.

^a^PSA density was not available in our database but was only used to distinguish very low and low-risk AUA and NCCN groups, who had no different recommendations in bone scan staging.

A high risk of bias was identified in all literature-derived models due to small sample sizes, limited internal and external validations and some biased recruitment processes (Supplementary Tables 4 and 5 and Supplementary Fig. 5). The rationale behind threshold selection for staging strategies was sometimes missing [30] or poor. Three main approaches were used to select thresholds: percent bone scan positivity (inadequate in small studies where observed risk may not generalize) [7], negative predictive value (insensitive for rare events) and the highest point on the ROC curve (balancing sensitivity and specificity equally though sensitivity must be higher in this context).

### Model validation

No model had the ideal calibration-in-the-large of zero (Table 3). Most models had a positive calibration-in-the-large, indicating they under-estimated risk on average. Lai deviated least in calibration-in-the-large (−0.28 [95% confidence interval, CI: −0.37, −0.19]) and Ho deviated most (−1.88 [95% CI: −1.96, −1.80]), overestimating risk on average.

**Table 3:** Calibration and discrimination of models.

Models	Calibration in the large (intercept) [95% CI]	Calibration slope [95% CI]	Discrimination as AUC [95% CI]
Continuous models			
Chybowski et al. 1991 [33]	0.84 [0.75, 0.93]	0.64 [0.59, 0.68]	0.75 [0.71, 0.79]
Ho et al. 2013 [31]	−1.88 [−1.96, −1.80]	1.80 [1.66, 1.94]	0.80 [0.75, 0.84]
Wang et al. 2013 [32]	0.76 [0.68, 0.85]	0.94 [0.88, 1.00]	0.79 [0.75, 0.83]
Categorical models			
Briganti et al. 2010 [4]	–	–	0.68 [0.64, 0.72]
Gnanapragasam-Cambridge Model	0.43 [0.35, 0.50]	1.36 [1.25, 1.46]	0.78 [0.74, 0.81]
ISUP Grade Grouping	0.56 [0.48, 0.64]	0.67 [0.61, 0.73]	0.72[0.68, 0.77]
Lai et al. 2011 [5]	−0.28 [−0.37, −0.19]	0.63 [0.58, 0.67]	0.74 [0.7, 0.77]
O’Sullivan et al. 2003 [34]	–	–	0.70 [0.67, 0.73]
Guidelines			
2018 AUA Guidelines [3]	–	–	0.74 [0.7, 0.77]
2020 EAU Guidelines [29]	–	–	0.73 [0.7, 0.77]
2019 NCCN Guidelines [30]	–	–	0.73 [0.7, 0.77]

Calibration statistics not given for Briganti or O’Sullivan models as they had three or fewer risk groups, precluding calculation of meaningful calibration statistics. Calibration statistics also could not be calculated for guideline models, which did not provide numeric estimates of risk in risk groups, precluding any calculation of calibration statistics (comparisons of numerical observed and predicted risk).

*AUC* Area under the receiver operator characteristics curve, *ISUP* International Society of Urological Pathology, *AUA* \American Urological Association, *EAU* European Association of Urology, *NCCN* National Comprehensive Cancer Network.

Calibration slope was also rarely one, the ideal (Table 3). The Wang model was closest with slope 0.94 [95% CI: 0.88, 1.00], but most others deviated significantly. Those with slope less than one (Chybowski, ISUP and Lai) over-predicted risk in high-risk groups and under-predicted it in low-risk groups (Supplementary Fig. 6), classic of overfitting. The Ho and Gnanapragasam models had slopes far greater than one. Their calibration plots suggest this was likely due to under-prediction of risk in high-risk groups for Gnanapragasam and over-prediction in low-risk groups for Ho (Supplementary Fig. 6).

Discrimination ranged from 0.68 to 0.80 for all models, considered “fair” by Hosmer et al. [24]. The highest AUCs were seen with Ho, Wang and Gnanapragasam (Table 3).

### Strategy validation

**Fig. 2:**
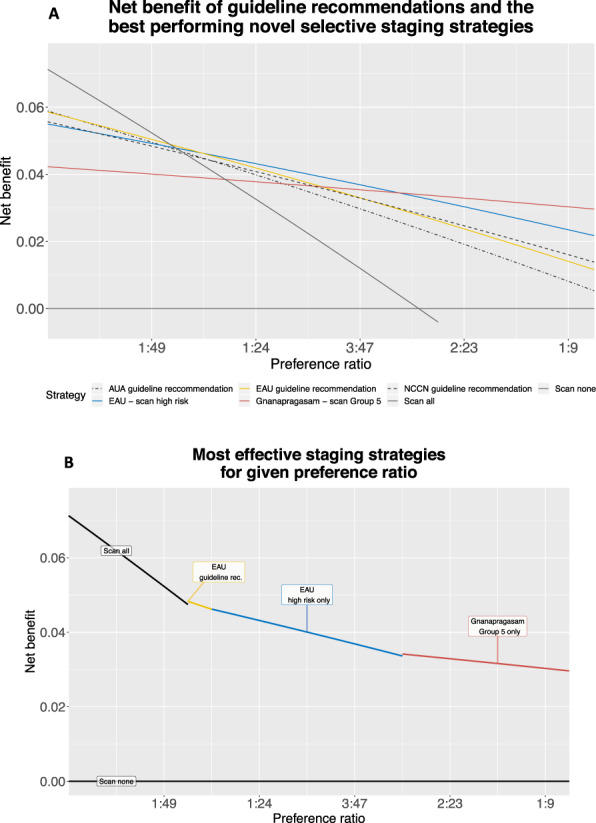
Model performance by decision curve analysis. **A** Decision analysis curves for guideline recommendations and top-performing alternative staging strategies. **B** Stepwise plot demonstrating optimal staging strategy for each potential preference ratio.

Figure 2 summarizes net-benefit comparisons. Part A presents the decision-analysis curves for guideline recommendations and the two novel selective staging strategies that superseded all other approaches: scanning EAU high risk patients only and Gnanapragasam Group 5 patients only. Part B highlights the strategy performing best at each assessed preference ratio. The EAU guideline recommendation was best for preference ratios 1:39–3:97 (NWT 40–33), scanning EAU high-risk patients for preference ratios 3:97 to 7:93 (NWT 32–14) and scanning Gnanapragasam Group 5 patients for preference ratios 7:93–1:9 (NWT 13–10). The scan-all strategy had higher net-benefit than all other strategies at preference ratios 1:99–1:39 (representing a number-willing-to-test to capture a positive scan, NWT, 100–40). Supplementary Fig. 7 has decision-analysis curves for all strategies.

Supplementary Table 7 presents net-benefit for each model’s recommended staging strategy (the strategy advised by the model’s source) at different preference ratios, above the net-benefit from the best performing strategy in that model for that preference ratio. There was often a discrepancy, indicating the benefit of using net-benefit to identify optimal staging strategies. The table also shows that fixed strategies from continuous models often had higher net-benefit than the continuous model itself at the same preference ratio. This may be due to mis-calibration.

## Discussion

Bone scans are the most widely available tool for prostate cancer staging and remain the most evidence-based in guiding treatment selection [2]. Bone scan results can significantly alter the optimal treatment plan for newly diagnosed prostate cancer patients. A finding of oligometastases may lead a patient from radical curative treatment to combined radiotherapy and systemic therapy, or from systemic to combined radiotherapy and systemic therapy. However, recommendations for bone scan staging vary and are based upon consensus opinion or models developed in small cohorts often with selective recruitment and limited rigorous external validation. Ours is the first study to validate such a broad range of bone scan staging strategies head-to-head in a large independent cohort using net-benefit.

We found that (i) none of the commonly used models or strategies were universally superior across preference ratios, and (ii) the optimal staging strategy varied with preference ratio. Selective staging strategies that performed best were the EAU 2020 guideline recommendations (scanning patients with intermediate-risk GS 4 + 3 disease or high-risk disease), scanning EAU high-risk patients only and scanning patients in Group 5 of the novel Gnanapragasam model. The choice between them depends upon the preference ratio of conservatism and tolerance appropriate to the local health system and a given patient’s case. As bone scan results can radically alter treatment, some clinicians and patients may prefer more conservative approaches like the EAU guideline recommendation. In other scenarios, with different patients or health systems, or in health crises, such changes in treatment or such generous scanning may not be feasible, necessitating more “tolerant” strategies-like scanning EAU high-risk patients or Gnanapragasam Group 5 patients only.

Interestingly, at high levels of conservatism, scanning everyone had greater net-benefit than currently available selective staging strategies. This may be a result of true misses with selective staging strategies. In our pre-imputation cohort, approximately 3% (35/1086) of patients with GS 6 disease on biopsy had positive staging bone scans. These patients are often excluded from selective staging strategies as GS 6 disease is often thought not to metastasize. However, upgrading of Gleason 6 prostate cancer is common on radical prostatectomy [37–39], and these patients may have a risk of metastatic disease higher than appreciated by current selective staging strategies. Additional predictors of final grade, like PIRADS score, may improve the accuracy of selective staging strategies at conservative preference ratios [40]. A scan-all approach may also have appeared superior to selective staging approaches because of false positives. Present literature suggests a 79% specificity of bone scan staging [41], but patients with low-risk disease were often excluded from these studies. Our own data suggest a higher rate of false-positive scans in patients with low-risk disease, as indeterminate scans in patients with low-risk disease were classified as negative more often than in patients with high-risk disease. False positives have the potential of inappropriately altering treatment plans and leading to sub-optimal care, and thus such inclusive strategies should be used with care. Improved imaging technologies should bring fewer false positives, and conditioning future models on true positive scan results rather than all positives could also circumvent this issue in future.

Our analysis confirmed inaccuracies in bone scan positivity risk prediction by current models. Ho and Gnanapragasam were overfitted (calibration slope more than one), and Chybowski, Lai and ISUP were underfitted (calibration slope less than one). Both are consequences of small sample sizes, having fewer than ten events (positive bone scans) per predictor-variable (EPV) at model development or few events at model validation and repurposing (ISUP and Gnanapragasam) [42]. Calibration issues are likely responsible for differences in net-benefit from continuous models and the “fixed strategies” recommended from them. Recalibration may prove these models more useful. This analysis confirms the widespread problems of model development noted by Moon et al. [42], but also shows that despite mis-calibration, the Gnanapragasam model provided a highly effective selective staging strategy, underscoring the importance of practical measures of model performance like net-benefit.

Another key strength of our study is it is one of few studies in this field to meet the sample size requirements for reliable external validation [42, 43]. Our cohort was also derived from an opt-out population-based registry, with minimal exclusion criteria, limiting selection bias. Although missing data is a key limitation, this is a common issue in this field [42], and our study is the first to report on it in such detail and the first in the field to use multiple imputation to handle it. Additionally, we have strong evidence to support the reliability of our imputations, with post-imputation distributions of prostate cancer disease characteristics fitting those expected in a prostate cancer population. Finally, while PSMA-PET use is extending to primary prostate cancer staging [44, 45], radionuclide bone scans have the most evidence in guiding treatment strategies and have FDA approval [2]. Thus, this work is of critical relevance and use now, and in future, may help evaluate PSMA-PET staging.

This study found that no single model performed best for selective bone scan staging, and rather different strategies from different models were better than others over different degrees of conservatism and tolerance. Of the selective staging strategies assessed, three performed best: scanning patients as per the 2020 EAU guideline, scanning EAU high-risk patients and scanning Gnanapragasam Group 5 patients. Scanning only EAU high-risk patients provided the greatest net-benefit over the greatest range of preference ratios (NWT 14–32), but other approaches may be preferred in different settings with different degrees of conservatism and tolerance. This study provides a robust analysis that can improve bone scan use and decision making now in primary prostate cancer staging, and acts as a flagship for the assessment of future technologies like PSMA-PET/CT.

## Supplementary information

Supplementary

## Data Availability

Analyses and imputations were performed using open-source code within the CRAN repository [26, 46–49]. Additional code required for data cleaning and incorporating multiply imputed data into the analysis was tailored to the dataset.
