# Supplementary material for: Evaluation of selective bone scan staging in prostate cancer – external validation of current strategies and decision-curve analysis
Source: Prostate Cancer Prostatic Dis. 2022 Mar 14;25(2):336–43. doi: 10.1038/s41391-022-00515-8 (PMC9184265; doi:10.1038/s41391-022-00515-8)
Supplement: Supplementary file 1 — Supplementary [file 41391_2022_515_MOESM1_ESM.pdf]

1 Supplementary 1

Bone scan data can be absent where a) a scan was not conducted or b) where data on the scan is not available. Reasons for missing bone scan data were explored by comparing characteristics of patients missing this data and patients with it. Supplementary Table 1 shows that patients with staging bone scans had higher grade disease, higher PSA, higher T stage and fewer positive cores on biopsy than those that did not –all factors routinely used to select patients for bone scan staging as per clinical guidelines(1-3). They were also more often private patients (64% v. 29%, Supplementary Table 1), likely reflecting data-access issues. These variables and survival help explain reasons for missing scan results and so were used in the multiple imputation equation to allow imputation under a missing-at-random assumption.

Bone scan imputation was informed by prostate cancer specific survival, initial treatment type, clinical and pathological T-stage, Gleason grade group, PSA and patient age. Imputation of missing predictor data (percentage positive cores, clinical T-stage, PSA, lymph node involvement, Gleason scores) were informed by these predictor variables: pathological T-stage, treatment type, patient survival, public or private treatment, each other and D'Amico risk strata (for all variables except PSA, T-stage and Gleason score). Ours is one of the first studies to address missing data in such detail, and tackle it with this level of rigor. Further, we find the summary of cohort characteristics post-imputation (Supplementary Table 2) close to population level data collected by SEER and other national databases (with rate of positive bone scans close to SEER estimated incidence of metastatic prostate cancer at diagnosis, 6.3%(4)). Furthermore, Kaplan-Meier survival curves (Supplementary Figure 1) show that those patients imputed to have positive bone scans had similar survival to those with known positive bone scans, and likewise for patients imputed with a negative bone scan. Survival (time to last contact, and event status at that time) was a fully known variable. These curves further cement the reliability of our imputations.

Supplementary Table 1 – Patient and disease characteristics at diagnosis compared between those missing a staging bone scan and those with a staging bone scan

		No staging BS on record	Staging BS
n		*6642*	*4079*
Age at diagnosis (mean (sd))		67.42 (8.96)	68.67 (9.01)
Publically treated patients (%)		2323 (35.6)	2716 (70.8)
ISUP Gleason Grade Group (%)	Group 1	2727 (42.2)	1090 (27.2)
	Group 2	1684 (26.1)	1093 (27.3)
	Group 3	928 (14.4)	768 (19.2)
	Group 4	611 (9.5)	515 (12.9)
	Group 5	511 (7.9)	537 (13.4)
PSA at diagnosis (median [IQR])		7.47 [5.36, 11.00]	9.70 [6.60, 17.00]
T stage at diagnosis (%)	T1	876 (71.9)	717 (55.7)
	T2	270 (22.2)	449 (34.9)
	T3	45 (3.7)	82 (6.4)
	T4	27 (2.2)	40 (3.1)
Percent positive cores on biopsy (median [IQR])		30.77 [16.00, 50.00]	41.67 [25.00, 66.67]
Initial treatment type (%)	Active surveillance	751 (14.1)	186 (4.9)
	Brachytherapy	349 (6.6)	311 (8.3)
	External Beam Radiotherapy*	1 007 (18.9)	1499 (39.8)
	Radical prostatectomy	2745 (51.6)	1234 (32.8)
	Systemic therapy (inc. ADT and chemotherapy)	222 (4.2)	409 (10.9)
	Watchful waiting	247 (4.6)	125 (3.3)
10 year survival (%) [95% CI]		93 [92, 94]	87 [86, 89]

BS = Bone scan, ISUP = International Society of Urological Pathology, PSA = Prostate Specific Antigen, IQR – Interquartile Range, ADT – Androgen Deprivation Therapy, CI = Confidence interval

*includes 109 patients who had palliative external beam radiotherapy, 81 of whom were missing a staging bone scan and 28 who had a staging bone scan on file

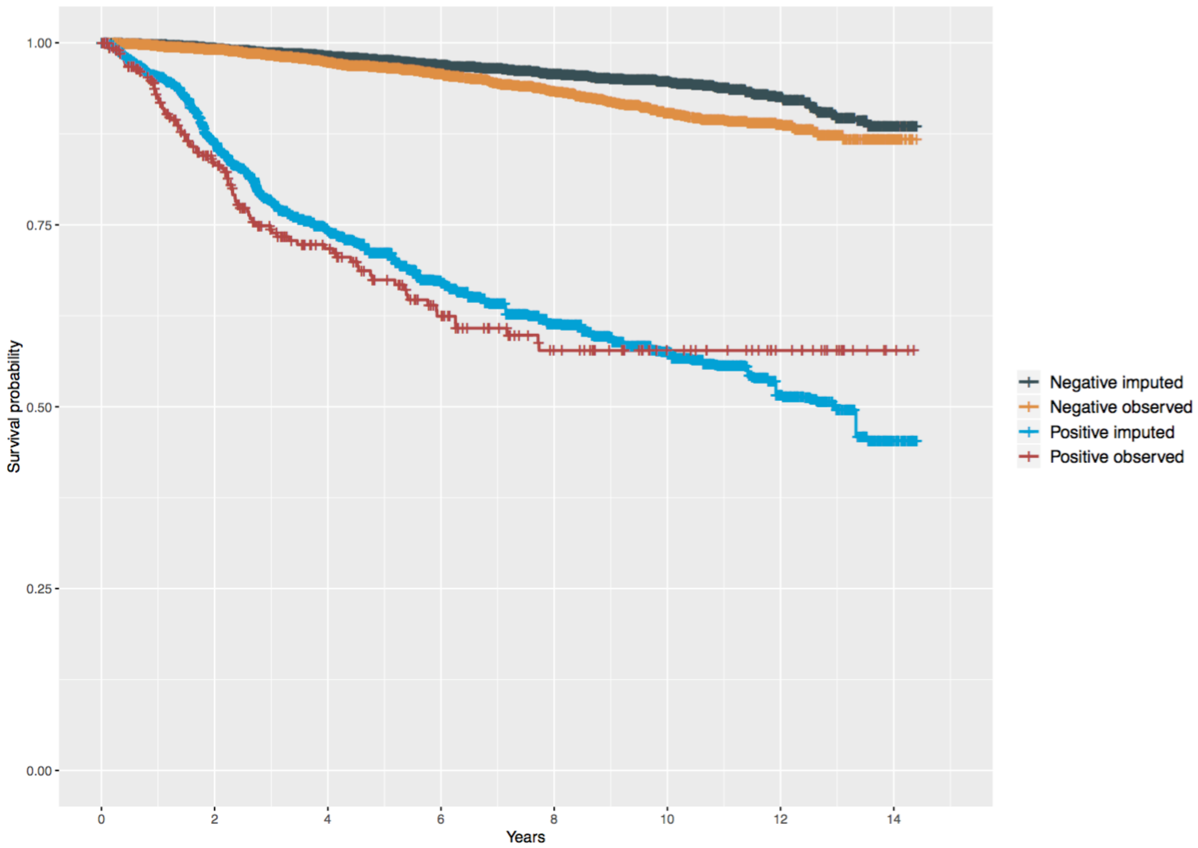

Supplementary Figure 1 – Survival of Patients by Staging Bone Scan Outcome: Imputed positive v. Observed positive v. Imputed negative v. Observed positive

1.1 Patients with staging bone scans on record and patients without staging bone scans on record

Ten-year survival was marginally better in patients without a staging bone scan on record, 93% [95% CI: 92 to 94] compared to 87% [95% CI: 86 to 89] (Supplementary Table 1) (see also Kaplan Meier Curve Supplementary Figure 2). This likely corresponds to a greater preponderance for lower risk disease in this group. 7% of patients without a staging bone scan on record fell into the D'Amico low risk group, compared to 5% of patients with a staging bone scan on record. Additionally, patients without staging bone scans on the system tended to have lower stage disease and lower Gleason Grade Group disease (Supplementary Table 1). This supports the belief that many people without staging bone scans on record, were actively excluded from bone scan staging because of perceived low risk disease. Furthermore, in subgroup analysis, survival was similar in patients with bone scan data on record and patients without bone scan data on record within low, intermediate and high D'Amico risk categories (Supplementary Figure 3). The loss of survival difference on stratification indicates the survival difference is largely driven by differences in the distribution of low, intermediate

and high-risk disease between patients with a staging bone scan on record and patients without a staging bone scan on record. Multivariate Cox Proportional Hazards modelling confirms this, demonstrating that once the different distributions of low, intermediate and high-risk disease are accounted for between these two groups, the statistical significance of the presence or absence of staging bone scan on record as a predictor of survival is lost (p = 0.0954) (Supplementary Table 2). To add to this, a small audit performed by our team on a stratified random sample of 89 patients without a staging bone scan on record revealed that 90% (45/50) of patients with low-risk disease truly did not undergo bone scan staging. This supports our use of factors like T-stage, Gleason Score and PSA as factors in explaining reasons for missingness in bone scan data, and feeding the multiple imputation model.

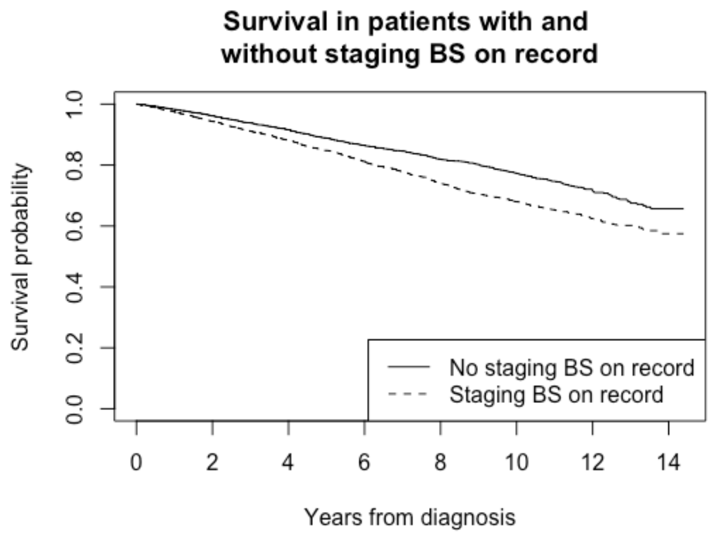

Supplementary Figure 2 - Survival based upon whether patient has a staging bone scan (BS) on record or not

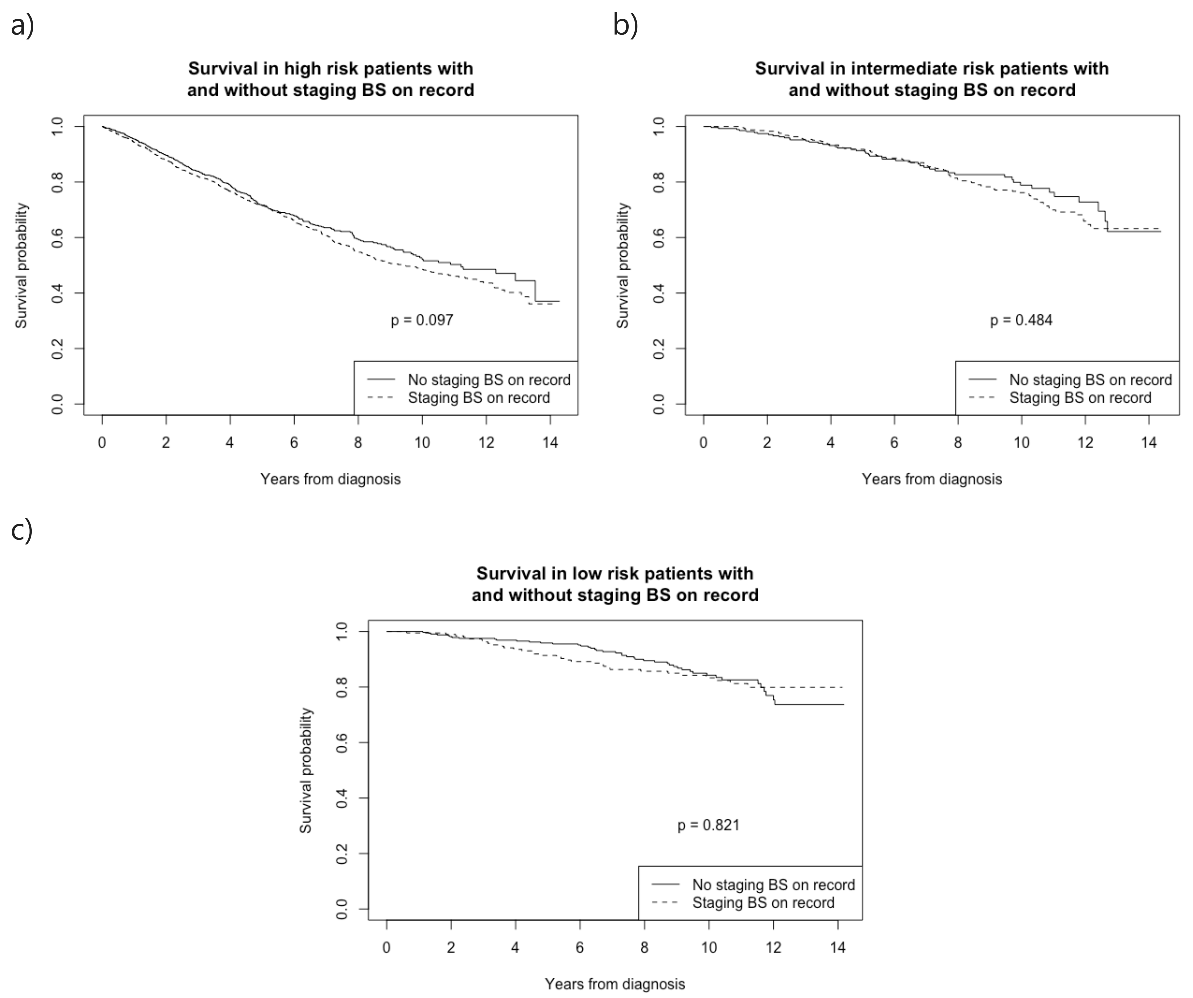

Supplementary Figure 3 - Survival within different risk strata based upon whether patient has a staging bone scan (BS) on record or not: a) high risk patients as per D'Amico classification, b) intermediate risk patients, c) low risk patients.

Supplementary Table 2 – Survival based on presence or absence of staging bone scan on record accounting for D'Amico risk group – A univariate and multivariate analysis

	Hazard ratios of Cox Proportional Hazards model [95% CI]		
	Univariate Analysis (Survival based upon presence/absence of staging bone scan on record)	Univariate Analysis (Survival based upon D'Amico risk stratification)	Multivariate Analysis (Survival based upon presence/absence of staging bone scan on record and D'Amico risk stratification)
Presence/absence of staging bone scan on record	1.45 [1.32, 1.58]*	-	1.11 [0.98, 1.26]
D'Amico intermediate-risk v. D'Amico high-risk	-	0.38 [0.32, 0.45]*	0.38 [0.32, 0.45]*
D'Amico low-risk v. D'Amico high-risk	-	0.24 [0.19, 0.31]*	0.25 [0.20, 0.31]*
*when reaching statistical significance at 0.05 level			

Supplementary Table 3 - Characteristics of model validation cohorts after 100 multiple imputations

	Median of imputation medians [IQR]
**Median Percentage of Positive Staging Bone Scans in Cohort [IQR]**	7.1 [6.8, 7.3]*
**Median Percentage of Clinically Node Positive Patients [IQR]**	6.7 [6.3, 7.0]
**Median Percentages with each ISUP Grade Group at Biopsy [IQR]**	
Grade Group 1	36.39 [36.36, 36.42]
Grade Group 2	26.48 [26.44, 26.51]
Grade Group 3	16.18 [16.15, 16.22]
Grade Group 4	10.79 [10.76, 10.82]
Grade Group 5	10.16 [10.13, 10.19]
**Median Percentages with each Clinical T stage [IQR]**	
T1	62.08 [61.78, 62.37]
T2	29.16 [28.85, 29.44]
T3	6.29 [6.08, 6.57]
T4	2.47 [2.41, 2.56]
**Median Percentage Positive Biopsy Cores [IQR]**	33.33 [33.33, 34.62]
**Median Diagnostic PSAs (ng/mL) [IQR]**	8.2 [8.2, 8.3]
**Median Percentages with each Pathological T stage [IQR]**	
pT1	15.68 [14.27, 16.92]
pT2	26.09 [25.21, 27.46]
pT3	49.02 [47.77, 50.07]
pT4	8.68 [7.89, 10.02]
**Median Percentages in each D'Amico Risk Group* [IQR]**	
Low risk	24.58 [24.33, 24.78]
Intermediate risk	42.48 [42.33, 42.70]
High risk	32.91 [32.65, 33.19]

ISUP = International Society of Urological Pathology, PSA = Prostate Specific Antigen, IQR = Interquartile Range

*From D'Amico et al. 2003

*Compared to 6.3% in SEER data(4)

2 Supplementary 2 – Model identification and Quality Assessment

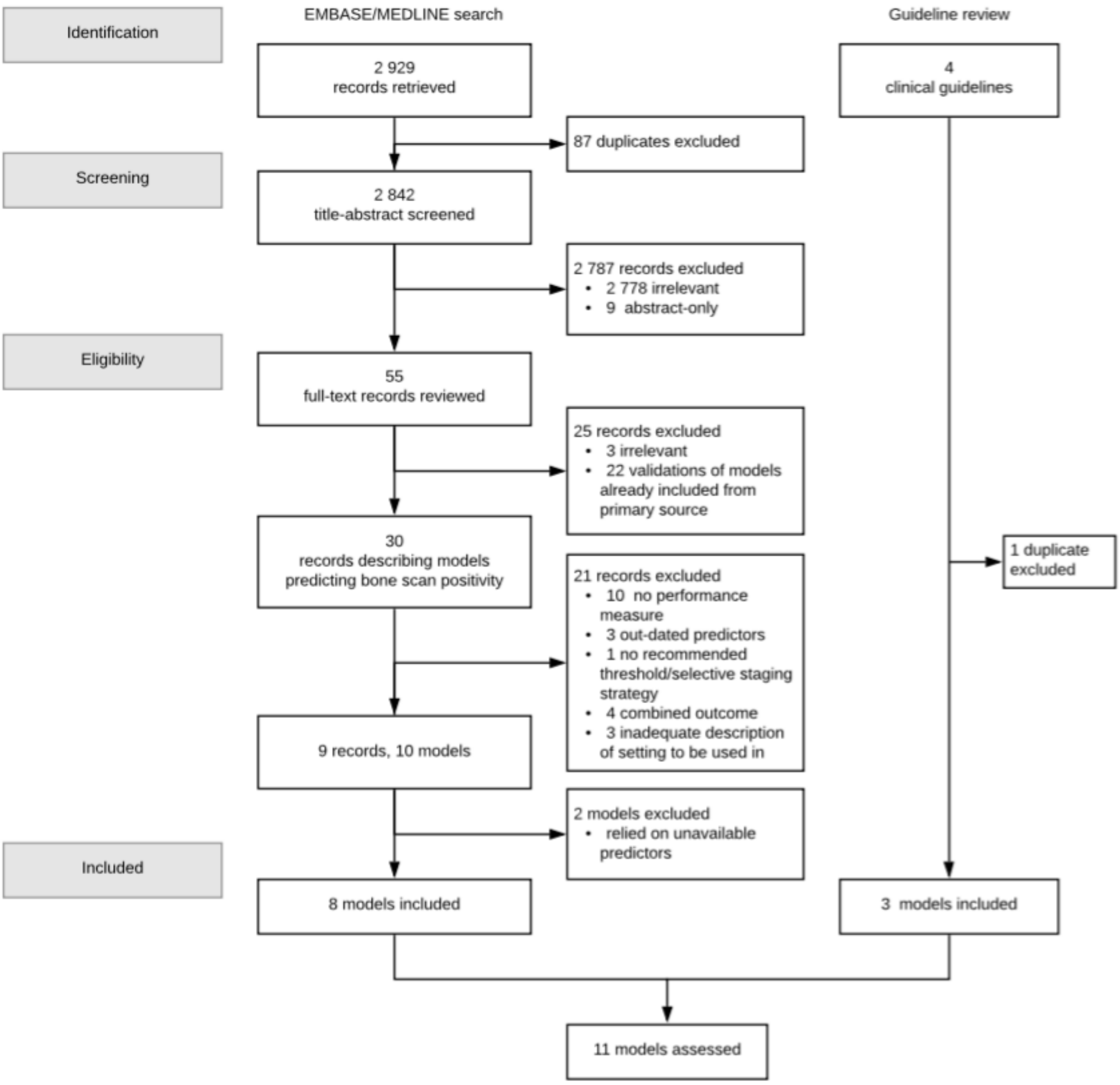

Supplementary Figure 4 – PRISMA flow chart for literature search and model selection. All 11 models were then validated in the SA-PCCOC cohort, obtaining their calibration, discrimination and decision analysis curves.

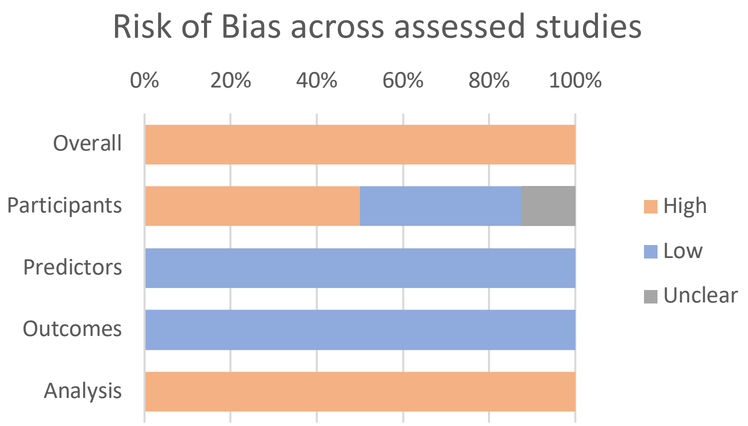

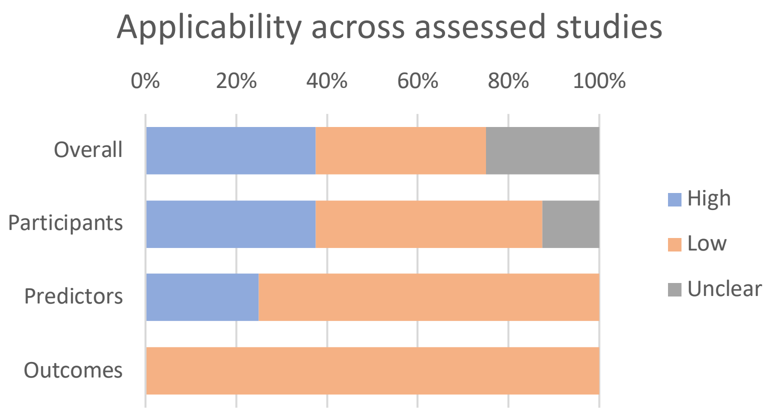

Supplementary Figure 5 – Summary of Risk of Bias and Applicability in the validated literature-derived models

Supplementary Table 4 – Performance on specific PROBAST domains for Risk of bias and applicability for each validated literature-derived model

Study	ROB				Applicability			Overall	
	Participants	Predictors	Outcomes	Analysis	Participants	Predictors	Outcomes	ROB	Applicability
**Chybowski**	+	+	+	-	+	+	+	-	+
**O'Sullivan**	+	+	+	-	+	+	+	-	+
**Briganti**	-	+	+	-	-	+	+	-	-
**Lai**	+	+	+	-	+	+	+	-	+
**Ho**	+	+	+	-	+	-	+	-	-
**Wang**	?	+	+	-	?	+	+	-	?
**Thurtle for Gnanapragasam**	-	+	+	-	-	-	+	-	-
**Thurtle for ISUP**	-	+	+	-	-	+	+	-	?

ROB = risk of bias, "+" indicates low ROB or low concern regarding applicability, "-" indicates high ROB or high concern regarding applicability, "?" indicates unclear ROB or unclear concern regarding applicability. Evaluated using PROBAST**(5)**

Supplementary Table 5 – Selection criteria, handling of missing data, sample size and other sources of bias

Tool	Number of Candidate Predictors*	EPV/Sample size	Recruitment strategy	Exclusion criteria	Data availability and handling of missing data	External validations
**Continuous**						
Chybowski et al, 1991**(6)**[†]	11<br>PSA<br>Acid Phosphatase<br>Prostatic Acid Phosphatase<br>Local clinical stage in 6 categories<br>Tumour grade in 4 categories	7	Randomly selected patients diagnosed at a single tertiary centre between 1987 and 1989	Any treatment prior to presentation at this centre (including hormonal manipulation)	Exploration: Describe missing information in one candidate predictor that has fallen out of use, but none in variables used in final model (i.e. none in bone scan, PSA, tumour stage or tumour grade data)<br><br>Handling: likely complete case analysis (exclusion of incomplete cases), but not explicitly described	Pal et al. 2008[15]; Chen et al., 1997[16]; Wolff et al., 1996[17]; Rudoni et al., 1995[18]
Ho et al, 2013**(7)**	9<br>PSA<br>Nodal status on cross-sectional imaging (CT or MRI) in two categories (N0 or N1)<br>Gleason score in 5 categories (≤5, 6, 7, 8, ≥9)<br>T-stage in three categories (T1, T2, ≥T3)	10	Consecutive patients presenting to one of two tertiary referral centres between January 2000 to May 2011 with a diagnosis of prostate cancer	Any other preexisting malignancy which may predispose them to bone metastasis<br><br>Presentation with acute urinary obstruction and had emergency prostate resection for relief of the obstruction<br><br>Patients who had pretreatment with antiandrogen/5- alpha reductase inhibitor (i.e. including hormonal therapy for BPH)	Exploration: Report 23 of 281 patients had missing data, but do not report in which variables their data was missing.<br><br>Handling: Report excluding patients with missing data i.e. complete case analysis	
Wang et al, 2013**(8)**	3<br>T-stage in two categories (<T4, T4)<br>Gleason score in two categories (≤3+4, ≥4+3)<br>PSA	22	Unclear if a consecutive, random or alternate recruitment strategy was used, but all patients had histologically confirmed prostate cancer diagnosed between January 2009 and December 2011 at a single tertiary centre	No explicit exclusion criteria stated	Exploration: No description of missing data<br><br>Handling: no description, suggesting complete case analysis and exclusion of patients with missing data	

Tool	Number of Candidate Predictors*	EPV/Sample size	Recruitment strategy	Exclusion criteria	Data availability and handling of missing data	External validations
**Discrete**						
Briganti et al, 2010**(9)**	8<br>PSA in four categories (0-4, 4-10, 10-20, >20)<br>T-stage in three categories (T1, T2, T3)<br>Gleason score in four categories (≤6, 3+4, 4+3, ≥8)	3	Consecutive patients with PCa diagnosed between January 2003 and June 2008 at a single tertiary referral centre (possibly only the surgical unit of this centre)	Note there were no patients with T4 disease in study, but there is no explicit exclusion criterion for T4 disease.<br><br>Report no patient was on hormonal therapy at the time of the staging imaging, but neither this nor other treatment prior to investigation are explicit exclusion criteria	Exploration: Report no missing data on patients recruited	De Nunzio et al., 2013(10); Tanaka et al., 2011(11); Lu et al., 2016(12); Chien et al., 2016(13)[#]
Gnanapragasam**(14, 15)** and ISUP**(14, 15)** models repurposed by Thurtle et al. 2018 ISUP[†] repurposed	Gnanapragasam model:<br>8<br>PSA as three categories (<10, 10-20, >20)<br>T-stage by mpMRI in three categories (T1-T2, T3, T4)<br>Gleason Grade Group in five categories (Groups 1 to 5)<br><br>ISUP model:<br>5<br>Gleason grade group (groups 1 to 5)	Sample size low by validation study standard (i.e. less than 100 positive scans*	Cases were prostate cancer patients referred to the radiology centre of this tertiary centre for bone scan staging between January 2010 to May 2015	Criteria for referral to radiology unit not described. Of note, while the cohort had patients with traditionally high and low and risk factors for bone scan positivity, high risk patients were relatively over-represented.<br><br>Criteria for selection not further described.	Exploration: No description of missing data<br><br>Handling: no description, suggesting complete case analysis and exclusion of patients with missing data	-
Lai et al, 2011**(16)**	4<br>PSA in four categories (≤10, 11-20, 21- 200, >200)<br>Gleason score (as continuous variable)	9	Consecutive patients diagnosed with prostate cancer on TRUS biopsy between 1st January 1997 and 31st December 2004, at a single secondary referral centre	Previous treatment including any prostatic surgery<br><br>All patients with prostatic symptoms were evaluated with TRUS biopsy, suggesting any incidental diagnoses on TURP	Exploration: No description of missing data<br><br>Handling: no description, suggesting complete case analysis and exclusion of patients with missing data	

Tool	Number of Candidate Predictors*	EPV/Sample size	Recruitment strategy	Exclusion criteria	Data availability and handling of missing data	External validations
				may not have been included (or were re-biopsied –unclear)		
O'Sullivan et al, 2003**(17)**	5<br>PSA in four categories (≤20ng/mL >20 ng/mL, ≤10ng/mL and >10 ng/mL)<br>T stage in 2 categories<br>Gleason score in 2 categories	13	Consecutive patients with prostate cancer who had a staging isotope bone scan at the Royal Marsden NHS Trust between 1 January 1995 and 31 December 2000	Hormonal therapy or definitive treatment at time of scanning<br><br>PSA taken >30 days after or before bone scan<br><br>PSA taken while on hormonal therapy<br><br>Histological diagnosis not confirmed at Royal Marsden Hospital	Exploration: Describe missing data and explore reasons for missing data.<br><br>Handling: Attempts to rectify missing data by audit, and where not possible make reasonable assumptions based on long term follow-up.	Ayyathurai, 2006(18)

EPV = events per candidate variable, rounded to nearest whole number, PSA = Prostate Specific Antigen, ISUP = International Society of Urological Pathology, BPH = Benign Prostatic Hyperplasia, mpMRI = multiparametric MRI, TRUS = Transrectal Ultrasound guided prostatic biopsy, TURP = Transurethral Resection of Prostate

*Candidate predictors = any variable considered for predicting bone scan risk in the model. Variables with multiple categories contribute n-1 candidate predictors to total sum.

[#]Because these models were generated elsewhere and these studies simply assess them for another purpose, the standard of appropriate sample size applied was that used for validation studies (events = 100), both still fall short of this minimum sample size requirement.

3 Supplementary 3 – Additional results

3.1 Calibration

3.1.1 Predicted probabilities of bone scan positivity in literature

Supplementary Table 6 – Predicted risk of bone scan positivity by risk category or regression equation in literature-derived models of bone scan positivity

Study	Predicted Probabilities				
**Continuous**					
Chybowski	Predicted using a graph relating probability of positive bone scans to PSA at diagnosis, displaying a logistic regression curve with PSA on a log scale. Measuring points on these axes, the curve was estimated to follow the equation: $logit(P) = -7.859 + 1.567 * log(PSA)$ where P = probability of a positive bone scan and PSA is measured in ng/mL				
Ho	Predicted using the following logistic regression equation: $logit(P) = -1.069 + 0.007 * PSA + 1.021 * Nodal\ status$ where P = probability of a positive bone scan, PSA is measured in ng/mL and Nodal status = 1 if suspicious nodes are present on cross-sectional imaging and 0 if not				
Wang	Predicted using the following logistic regression equation: $logit(P) = -6.40 + 2.39 * Tstage4 + 0.87 * log(PSA + 1) + 0.93GS$ where P = probability of a positive bone scan, PSA is measured in ng/mL, TStage4 = 1 if disease T4 and 0 if not, and GS = 1 if Gleason Score 4+3 or higher and 0 if not				
**Discrete**					
Briganti	Low risk:<br>3/692 i.e. approx. 0.4%	Intermediate risk:<br>6/72 i.e. approx. 8.3%	High risk:<br>15/89 i.e. 16.9%		
Thurtle for Gnanapragasam	Group 1:<br>0/10 i.e. 0%	Group 2:<br>0/40 i.e. 0%	Group 3:<br>1/47 i.e. 2.1%	Group 4:<br>8/137 i.e. 5.8%	Group 5:<br>28/204 i.e. 13.7%
Thurtle for ISUP	Grade Group 1:<br>0/43 i.e. 0%	Grade Group 2:<br>1/109 i.e. 0.9%	Grade Group 3:<br>7/74 i.e. 9.6%	Grade Group 4:<br>9/91 i.e. 9.9%	Grade Group 5:<br>20/121 i.e. 16.5%
Lai	Group 1 (PSA 0-10):<br>0/25 i.e. 0%	Group 2 (PSA 11-20):<br>2/21 i.e. 9.5%	Group 3 (PSA 21-200):<br>15/53 i.e. 28.3%	Group 4 (>200):<br>17/17 i.e. 100%	
O'Sullivan	Patients not recommended for scanning:<br>1/130 i.e. 0.8%	Patients recommended for scanning:<br>67/290 i.e. 23.1%			

PSA = Prostate Specific Antigen, ISUP = International Society of Urological Pathology

Calibration plots for validated models

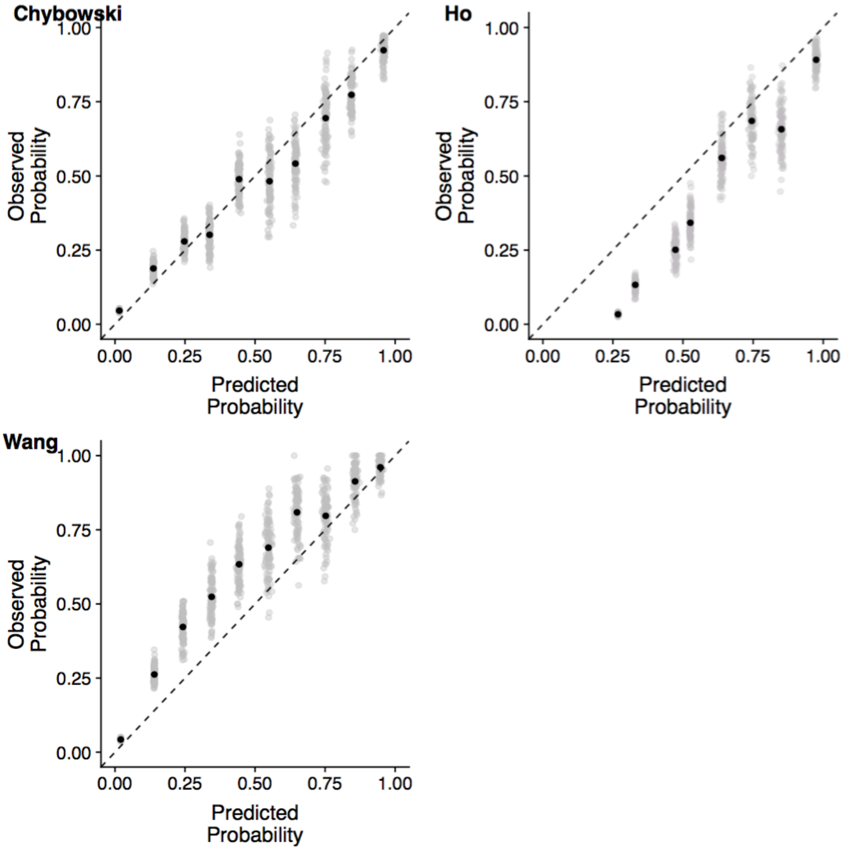

Supplementary Figure 6a – Calibration plots from external validation of continuous models predicting bone scan positivity risk

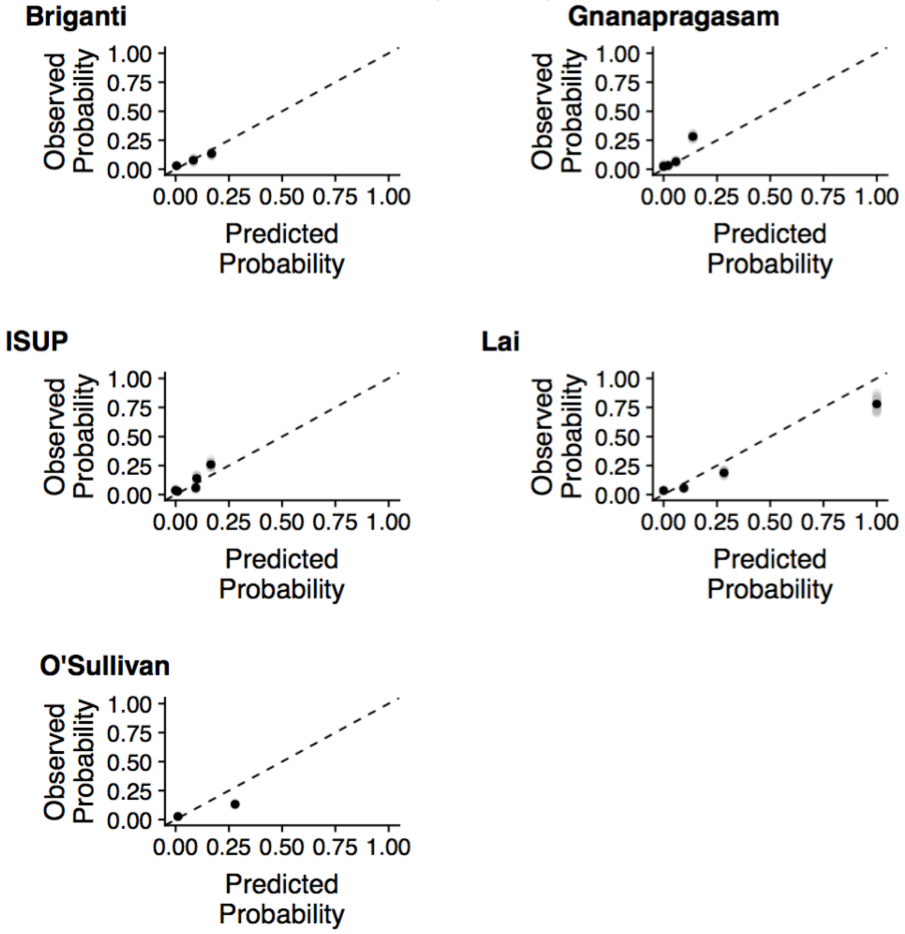

Supplementary Figure 6b – Calibration plots from external validation of discrete models predicting bone scan positivity risk

3.2 Net-benefit

3.2.1 Continuous models

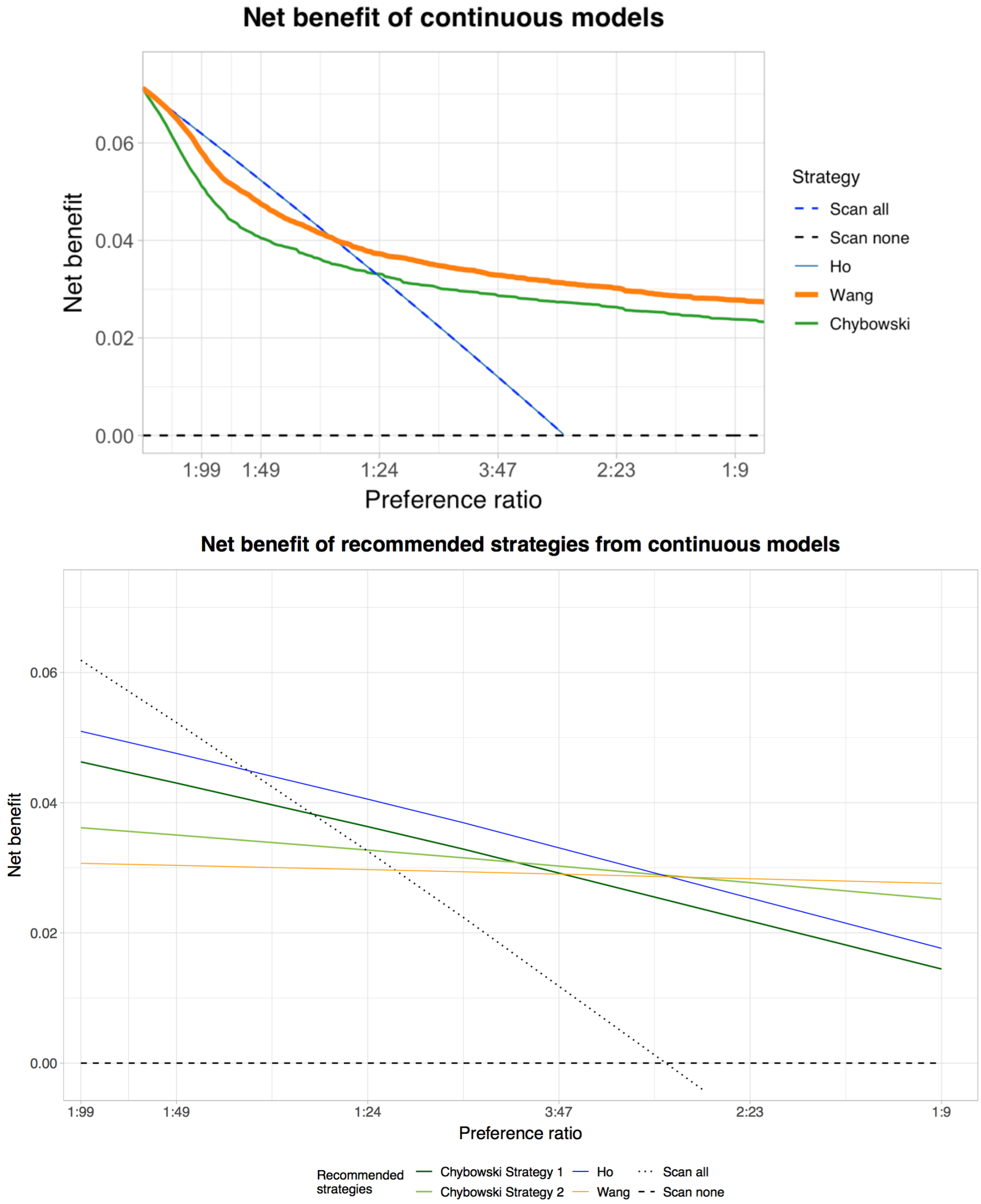

Supplementary Figure 7a – Decision analysis curves for continuous models derived from literature i) Net-benefit at each threshold of continuous models ii) Net benefit at recommended thresholds in continuous models

3.2.2 Discrete models

3.2.2.1 Methodology used to calculate net-benefit for categorical models and fixed strategies

Unlike selective staging strategies from continuous models, which vary with threshold probability (p_t), strategies from categorical models are less fluid and can only vary with the categories of the models. Thus, a change in p_t does not necessarily trigger a change in selection strategy in categorical models, like it does in continuous models, even though it

might change net-benefit. We felt this was best represented graphically by straight lines, as generated by Vicker's et al in their article describing interpretation of decision curve analysis and depicting the differences between decision curves for continuous models and "tests"(19). The equation for this line is given by:

$$\frac{TP}{N} - \left(\frac{p_t}{1 - p_t}\right)\frac{FP}{N}$$

where N is the total number of patients in the cohort, TP is the number of true positive bone scans identified by scanning patients in that category and above, FP is the number of people scanned in these categories who return a negative result (false positives), and p_t is the threshold probability at that given point in the line. This is taken from Vickers and Elkin's paper initially proposing net-benefit as a tool for evaluating model performance(20).

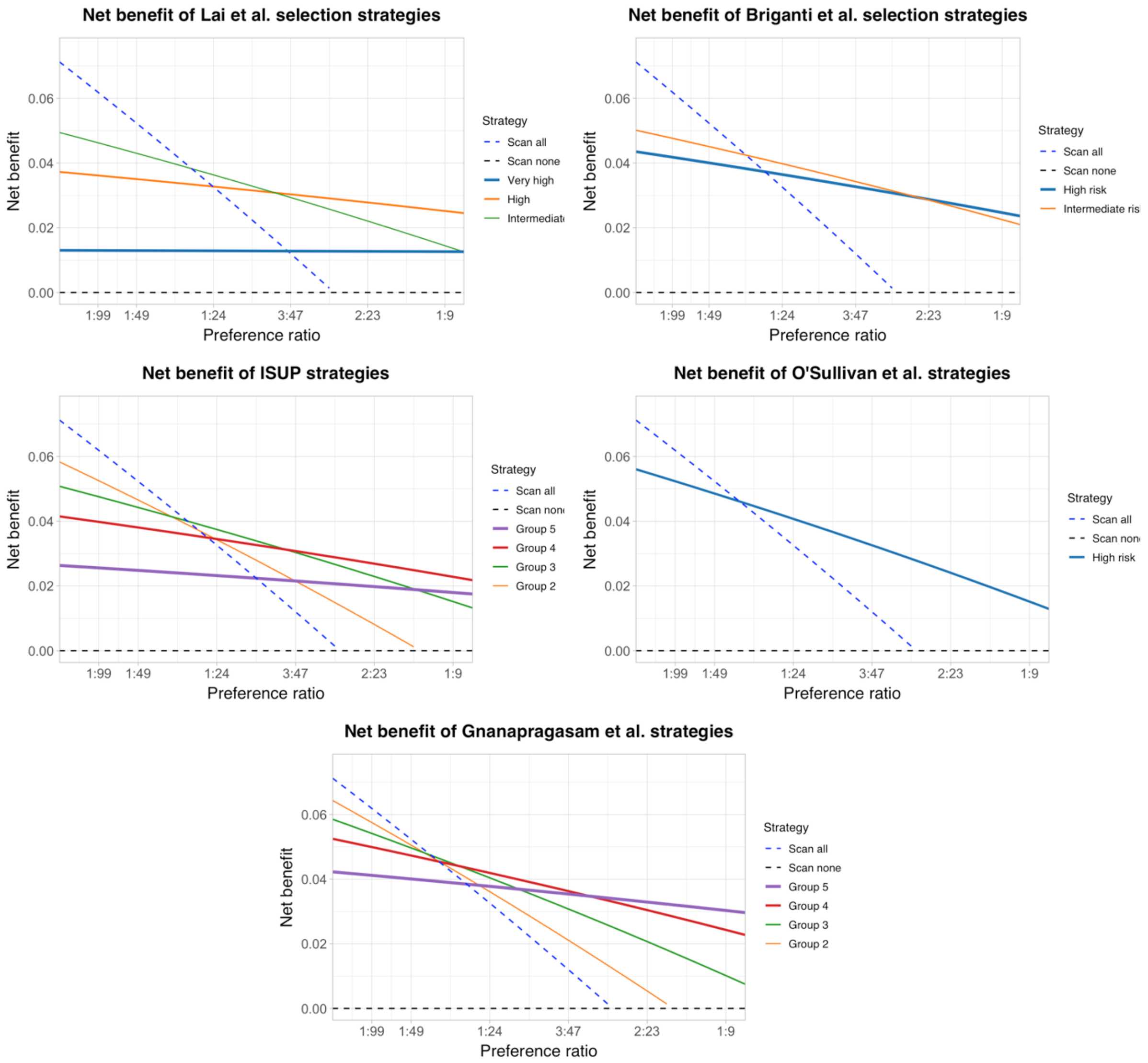

Supplementary Figure 7b – Decision analysis curves for discrete models derived from literature

3.2.3 Guidelines

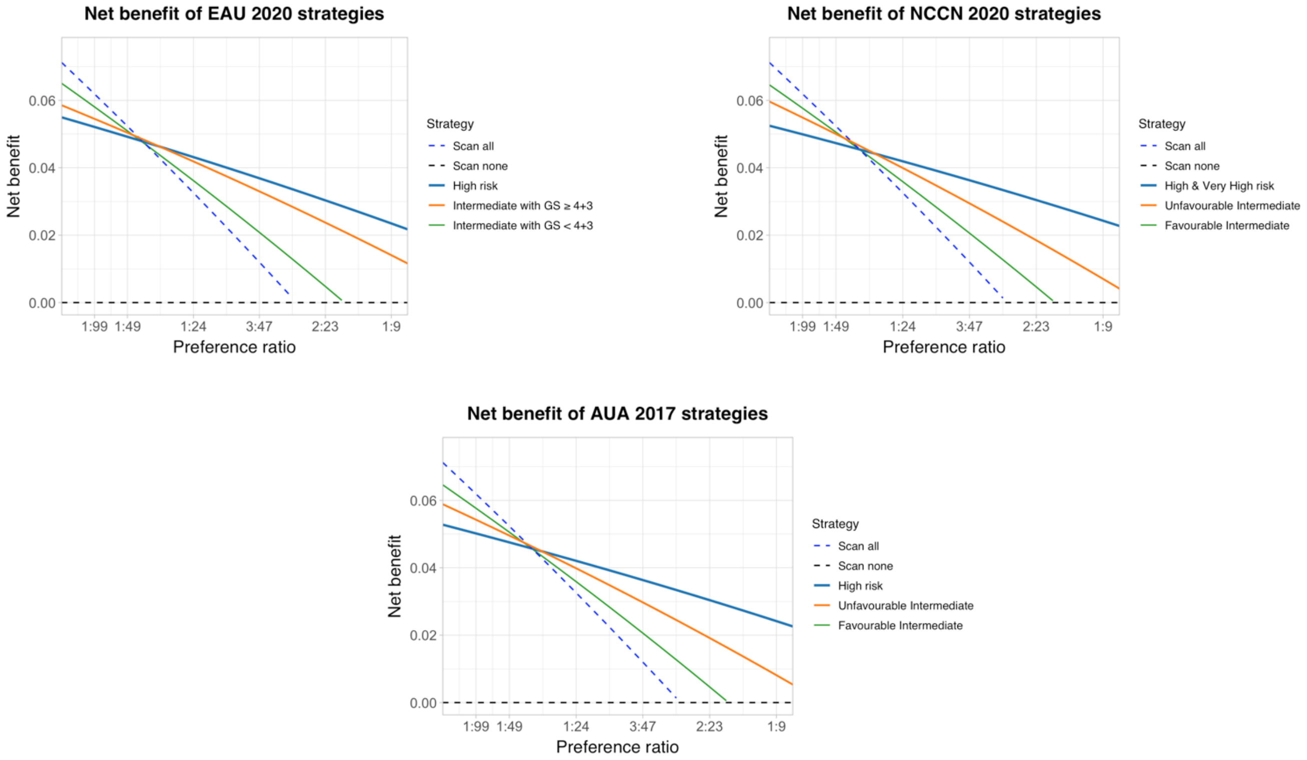

Supplementary Figure 7c – Decision analysis curves for clinical guidelines

3.2.1 Data table for net-benefit with different staging strategies

Supplementary Table 7 presents net-benefit for staging strategies from each model -those recommended from the model's source and the alternate ones available at different thresholds within the model. Listed under net-benefit for each categorical model's recommended strategy is the net-benefit achieved by the highest performing strategy in that model at that preference ratio. Listed under net-benefit of fixed strategies from continuous models (scanning people with PSA >10 in the Chybowski model, for example) is the net-benefit of the continuous model itself at the preference ratio. This uses the probability corresponding to the preference ratio (1:99 is p_t 0.01 for example) as the model's threshold for scanning(19).

Supplementary Table 7 – Net-benefit of staging strategies at different preference ratios

Model	Scanning Strategy	Net-benefit (and threshold for best strategy) at following preference ratios					
		1:99		1:24		1:9	
	Scan all	0.0619		0.0326		-0.0319	
EAU 2020	Int. risk w/ GS ≥ 4+3	0.0545		0.0419		0.0141	
	Best strategy in model	0.0619	(Low[3])	0.0432	(High)	0.0235	(High)
NCCN	Unfav. Int. risk and higher, or PSA > 20 & ≥T2	0.0549		0.0399		0.007	
	Best strategy in model	0.0619	(Low[3])	0.0419	(High & Very High)	0.0243	(High & Very High)
AUA	Unfav. Int. risk and higher	0.0542		0.0398		0.0081	
	Best strategy in model	0.0619	(Low[3])	0.0421	(High)	0.0242	(High)
Gnanapragasam	Scan ≥ Group 3	0.0541		0.0404		0.0102	
	Scan ≥ Group 4	0.0499		0.0419		0.0243	
	Best strategy in model	0.0619	(Group 1[3])	0.0419	(Group 4)	0.0303	(Group 5)
O'Sullivan	PSA >20, T4 or GS≥ 4+3	0.0523		0.0407		0.0152	
	Best strategy in model	0.0619	(Low[3])	0.0407	(High)	0.0152	(High)
Briganti	GS ≥8 or PSA >10 if T2-3[1]	0.0476		0.0398		0.0225	
	Best strategy in model	0.0619	(Low[3])	0.0398	(Intermediate)	0.0247	(High)
ISUP	Grade Group ≥ 3	0.0476		0.0374		0.0152	
	Best strategy in model	0.0619	(Group 1[3])	0.0374	(Group 3)	0.0228	(Group 4)
Lai	PSA > 10	0.0468		0.0364		0.0135	
	Best strategy in model	0.0619	(Low[3])	0.0363	(Intermediate)	0.0252	(High)
Chybowski	PSA > 10	0.0463		0.0363		0.0145	
	PSA >20	0.0362		0.0327		0.0252	
	From continuous model	0.0511	(pred. pr. <0.01)	0.0331	(pred. pr. <0.04)	0.0238	(pred. pr. <0.1)
Ho	PSA > 10 or cN1	0.051		0.0405		0.0176	
	From continuous model	0.0619	“[3]	0.0326	“	-0.0319	“
Wang	D < 0 using Wang eq.[2]	0.0307		0.0297		0.0276	
	From continuous model	0.058	“[3]	0.0373	“	0.0278	“

[1]Study excluded T4 disease, assumed T4 disease would be scanned, [2]D = –6.40 + 2.39Tstage4 + 0.87 ln(PSA+1) + 0.93GS + 2.169, where Tstage4 = 1 if T4 or 0 if less, and GS = 1 if Gleason score ≥4+3 or 0 if less [3]Equivalent of scanning all

Supplementary References:

1. Sanda MG, Chen RC, Crispino T, Freedland S, Greene K, Klotz LH, et al. CLINICALLY LOCALIZED PROSTATE CANCER: AUA/ASTRO/SUO GUIDELINE. 2017.
2. Mottet N, van den Bergh RCN, Briers E, Cornford P, De Santis M, Fanti S, et al. EAU - ESTRO - ESUR - SIOG Guidelines on Prostate Cancer 2020. European Association of Urology Guidelines 2020 Edition. presented at the EAU Annual Congress Amsterdam 2020. Arnhem, The Netherlands: European Association of Urology Guidelines Office; 2020.
3. Mohler JL, Antonarakis ES, Armstrong AJ, D'Amico AV, Davis BJ, Dorff T, et al. Prostate Cancer, Version 2.2019, NCCN Clinical Practice Guidelines in Oncology. 2019;17(5):479.
4. Surveillance Research Program. SEER*Explorer: An interactive website for SEER cancer statistics - 2021 [updated 12 July 2021. Available from: https://seer.cancer.gov/explorer/.
5. Moons KW, K; Riley, R; Whiting, P; Westwood, M; Collins, G; Reitsma, J; Kleijnen, J; Mallett, S. PROBAST: A Tool to Assess the Risk of Bias and Applicability of Prediction Model Studies. Annals of internal medicine. 2019;170(1):51-8.
6. Chybowski FM, Keller JJ, Bergstralh EJ, Oesterling JE. Predicting radionuclide bone scan findings in patients with newly diagnosed, untreated prostate cancer: prostate specific antigen is superior to all other clinical parameters. The Journal of urology. 1991;145(2):313-8.
7. Ho CC, Seong PK, Zainuddin ZM, Abdul Manaf MR, Parameswaran M, Razack AH. Retrospective study of predictors of bone metastasis in prostate cancer cases. Asian Pacific journal of cancer prevention : APJCP. 2013;14(5):3289-92.
8. Wang Y, Guo J, Xu L, Zhao N, Xu Z, Wang H, et al. Should bone scan be performed in Chinese prostate cancer patients at the time of diagnosis? Urologia internationalis. 2013;91(2):160-4.
9. Briganti A, Passoni N, Ferrari M, Capitanio U, Suardi N, Gallina A, et al. When to perform bone scan in patients with newly diagnosed prostate cancer: external validation of the currently available guidelines and proposal of a novel risk stratification tool. European urology. 2010;57(4):551-8.
10. De Nunzio C, Leonardo C, Franco G, Esperto F, Brassetti A, Simonelli G, et al. When to perform bone scan in patients with newly diagnosed prostate cancer: external validation of a novel risk stratification tool. World journal of urology. 2013;31(2):365-9.
11. Tanaka N, Fujimoto K, Shinkai T, Nakai Y, Kuwada M, Anai S, et al. Bone scan can be spared in asymptomatic prostate cancer patients with PSA of <=20 ng/ml and Gleason score of <=6 at the initial stage of diagnosis. Japanese journal of clinical oncology. 2011;41(10):1209-13.
12. Lu YM, Chien TM, Ke HL, Huang SP, Huang CN. The most suitable guidelines for performing bone scans in prostate cancer staging – One southern Taiwan medical center's results. Urol Sci. 2016;27(4):208-11.
13. Chien TM, Lu YM, Geng JH, Huang TY, Ke HL, Huang CN, et al. Predictors of Positive Bone Metastasis in Newly Diagnosed Prostate Cancer Patients. Asian Pacific journal of cancer prevention : APJCP. 2016;17(3):1187-91.
14. Thurtle D, Hsu RC, Chetan M, Lophatananon A, Hubbard R, Gnanapragasam VJ, et al. Incorporating multiparametric MRI staging and the new histological Grade Group system improves risk-stratified detection of bone metastasis in prostate cancer. British journal of cancer. 2016;115(11):1285-8.

15. Gnanapragasam VJ, Lophatananon A, Wright KA, Muir KR, Gavin A, Greenberg DC. Improving Clinical Risk Stratification at Diagnosis in Primary Prostate Cancer: A Prognostic Modelling Study. PLoS Medicine. 2016;13(8):e1002063.
16. Lai MH, Luk WH, Chan JC. Predicting bone scan findings using sPSA in patients newly diagnosed of prostate cancer: feasibility in Asian population. Urologic oncology. 2011;29(3):275-9.
17. O'Sullivan JM, Norman AR, Cook GJ, Fisher C, Dearnaley DP. Broadening the criteria for avoiding staging bone scans in prostate cancer: a retrospective study of patients at the Royal Marsden Hospital. BJU international. 2003;92(7):685-9.
18. Ayyathurai R, Mahapatra R, Rajasundaram R, Srinivasan V, Archard NP, Toussi H. A study on staging bone scans in newly diagnosed prostate cancer. Urologia internationalis. 2006;76(3):209-12.
19. Vickers AJ, van Calster B, Steyerberg EW. A simple, step-by-step guide to interpreting decision curve analysis. Diagn Progn Res. 2019;3:18.
20. Vickers AJ, Elkin EB. Decision curve analysis: a novel method for evaluating prediction models. Med Decis Making. 2006;26(6):565-74.
